# Facial Nerve Paralysis after Onyx Embolization of a Jugular Paraganglioma: A Case Report with a Long-Term Follow Up

**DOI:** 10.3390/jcm7030048

**Published:** 2018-03-07

**Authors:** Haitham Odat, Khaled Alawneh, Mohannad Al-Qudah

**Affiliations:** 1 Division of Otolaryngology, Department of Special Surgery, King Abdullah University Hospital, Faculty of Medicine, Jordan University of Science and Technology, PO Box 3030, Irbid 22110, Jordan; malqudah@just.edu.jo; 2 Department of Radiology, King Abdullah University Hospital, Faculty of Medicine, Jordan University of Science and Technology, PO Box 3030, Irbid 22110, Jordan; kzalawneh0@just.edu.jo

**Keywords:** jugular paraganglioma, jugular foramen, Onyx embolization, facial nerve paralysis

## Abstract

Jugular paragangliomas are slow growing highly vascular tumors arising from jugular paraganglia. The gold standard of treatment is complete surgical resection. Pre-operative embolization of these highly vascular tumors is essential to reduce intra-operative bleeding, allow safe dissection, and decrease operative time and post-operative complications. Onyx (ethylene-vinyl alcohol copolymer) has been widely used as permanent occluding material for vascular tumors of skull base because of its unique physical properties. We present the case of a 33-year-old woman who had left-sided facial nerve paralysis after Onyx embolization of jugular paraganglioma. The tumor was resected on the next day of embolization. The patient was followed up for 30 months with serial imaging studies and facial nerve assessment. The facial verve function improved from House–Brackmann grade V to grade II at the last visit.

## 1. Introduction

Jugular paragangliomas (JPs) are slow-growing highly vascular tumors that can cause extensive invasion of the surrounding vital structures [1]. The gold standard of treatment is complete surgical resection with pre-operative embolization [2]. When the tumor is inoperable, or the patient is unfit for surgery, radiotherapy or superselective embolization can be alternative primary treatment options [3].

Since 2005, ethylene-vinyl alcohol copolymer (Onyx; ev3 Neurovascular, Irvine, CA, USA) has been widely used as permanent occluding material for vascular tumors because of its unique physical properties [4].

Although Onyx is relatively safe and effective material for the occlusion of target vessels, cranial nerves deficit has been reported [5]. The stylomastoid and petrosal arteries are the major blood supply of the facial nerve (FN). The intimate relationship and shared blood supply between JP and the FN should be kept in mind during embolization [3].

We report a case of FN paralysis following Onyx embolization of JP and the outcome after the long follow-up period.

## 2. Case Presentation

**Figure 1:**
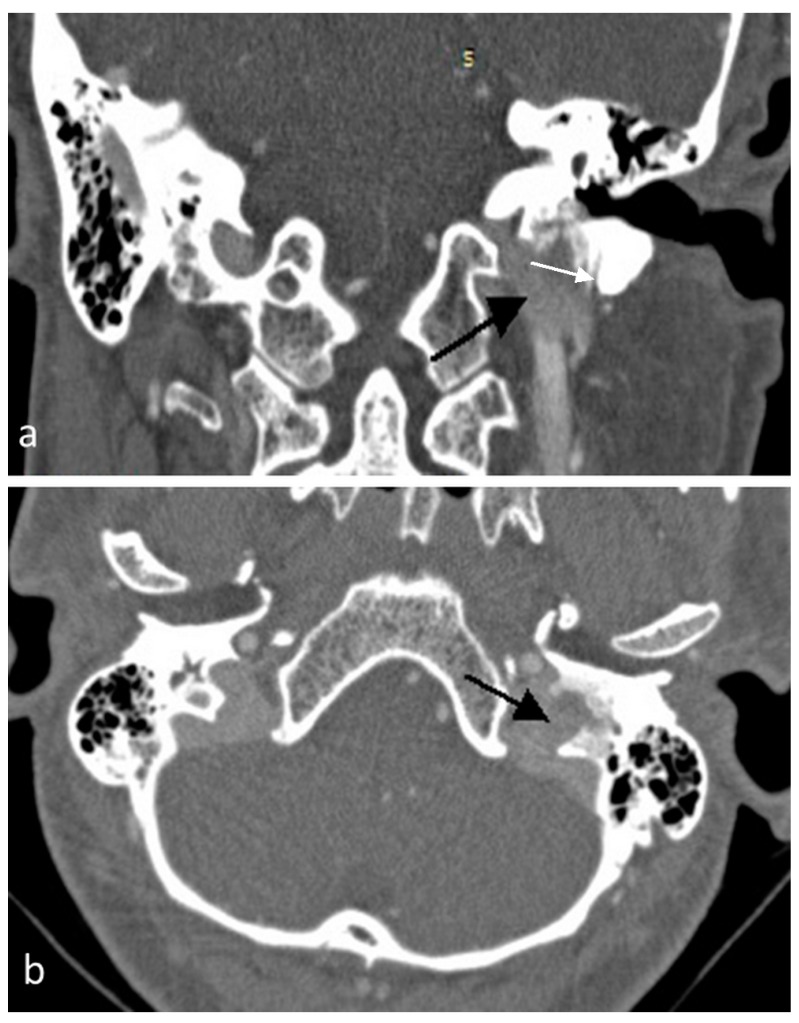
Computed tomography scan of temporal bone, (**a**) coronal and (**b**) axial views show soft tissue mass destructing the jugular foramen (black arrow). White arrow indicates stylomastoid foramen.

A 33-year-old woman presented to the otolaryngology clinic with left-sided pulsatile tinnitus and hearing loss for 18 months. Microscopic ear examination showed a bluish mass in the hypotympanic region. The facial nerve and lower cranial nerves were intact. Computed tomography (CT) revealed left soft tissue mass with irregular destruction of the jugular foramen (Figure 1) and heterogeneous isointense mass with salt-and-pepper appearance on magnetic resonance imaging (MRI). Radiological appearance was consistent with jugular paraganglioma (Fisch class C1). Surgical resection with pre-operative embolization was planned after modality of treatment was discussed with the patient.

Angiography revealed that the tumor was supplied by left postauriclar and middle meningeal arteries. Under general anesthesia, the left femoral artery was accessed. The external carotid artery was accessed using a guide wire and catheter, then superselective catheterization of each feeding artery was done by a micro-wire and Apollo^™^ detachable tip micro-catheter (ev3 Neurovascular, Irvine, CA, USA) followed by Onyx embolization. Postembolization angiogram demonstrated no contrast flow in the feeding vessels and complete tumor devascularization.

**Figure 2:**
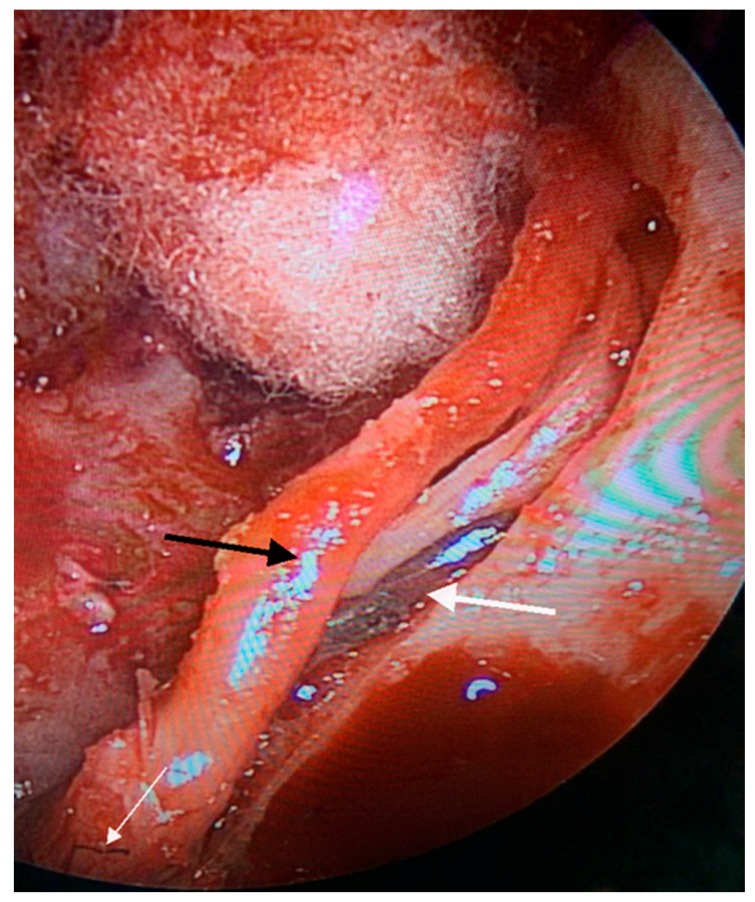
Endoscopic view of left ear shows the facial nerve mobilized from its canal (black arrow); the stylomastoid artery filled with Onyx (thick white arrow); Vasa nervosa filled with Onyx (thin white arrow).

The patient developed House-Brackmann (H-B) grade V FN paralysis immediately after embolization with Onyx, with no lower cranial nerves deficits. After 24 h, surgery through infratemporal fossa type A approach with temporary long anterior rerouting of the FN was performed. Intra-operatively, Onyx was seen filling the stylomastoid artery and scattered segments of the vasa nervosa of the FN (Figure 2).

During rerouting of the FN, some bleeding points were noticed which may indicate incomplete devascularization. Following gross total resection of the tumor, the FN was returned to its anatomical position (Brackmann modification). The patient had smooth recovery with normal lower cranial nerves function and was discharged home after 5 days.

The patient was followed-up regularly by serial CT, MRI, and FN assessment. After 5 months, the FN paralysis improved to grade IV, and at 18 months she had grade II, which stabilized until the last follow-up visit of 30 months.

**Figure 3:**
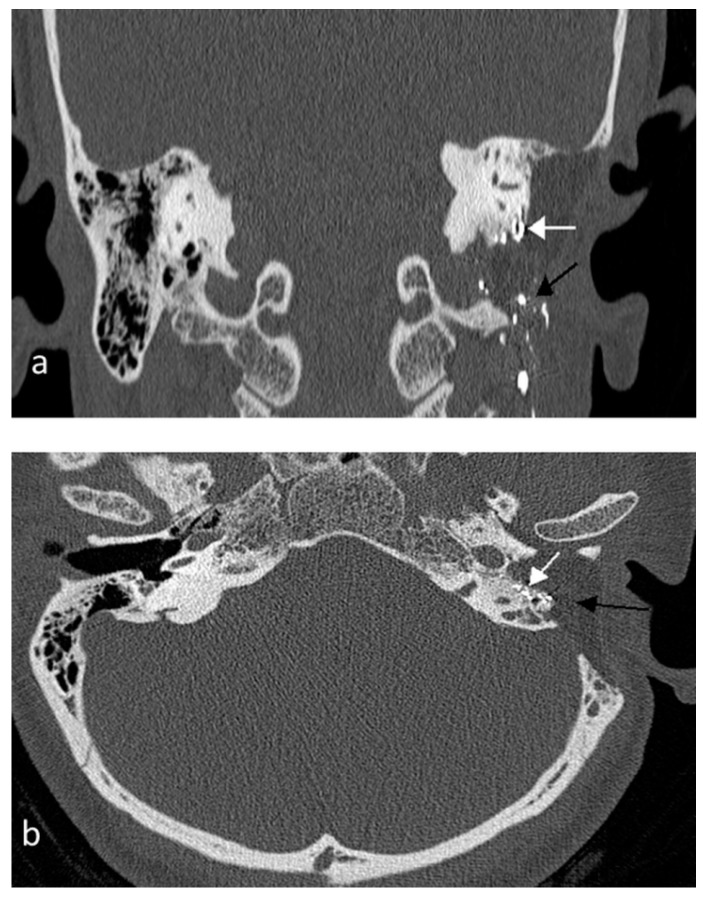
Post-operative computed tomography scan of temporal bone. (**a**) Coronal view shows hyper intense embolic material in the infralabrynthine (white arrow) and stylomastoid regions (black arrow). (**b**) Axial view shows middle ear and mastoid cavities filled with abdominal fat (black arrow); hyper intense embolic foci in the region of mastoid segment of the facial nerve (white arrow).

Her final CT and MRI after 30 months showed hyperintense areas of Onyx scattered in the middle ear and mastoid, with no recurrence of the rumor (Figure 3).

## 3. Discussion

Onyx is a permanent embolization material which has the property of reflux from the injected artery into other feeders with no need to do multiple embolizations [6]. It also has a delayed solidification time, which makes the risk of microcatheter adhesions low [7]. However, migration of Onyx to arteries supplying cranial nerves can cause neuropathy complications [8].

The intimate anatomical location and overlapped blood supply between the facial nerve and JP may explain the risk of post-embolization FN paralysis [5]. The true incidence of FN paralysis following embolization of JP with Onyx has not been established, and only few cases have been reported in the literature (Table 1). Gaynor and Michelozzi et al. [8,9] reported an incidence of 18% and 16% FN paralysis post-Onyx embolization, respectively; in our small series of six JP patients, one patient (17%) had FN paralysis.

In all reported cases, patients with complete FN paralysis (H-B grade VI) never had recovery, while all patients with partial paralysis after embolization showed improvement of their FN function and some had complete function recovery [9,10]. Our patient got partial FN function improvement from H-B grade V to II at the last follow-up visit. The partial recovery might be due to partial compensation of viable intrinsic arterial plexus of the FN for extrinsic blood supply interruption, as proposed by Odat et al. [2]. However, residual paresis might be explained by prolonged ischemia caused by Onyx or anterior rerouting of the FN itself which deprives the nerve from its extrinsic blood supply.

Our patient had surgery on the day following embolization; the FN was decompressed and temporarily anteriorly rerouted as part of the procedure for tumor resection. The four cases reported by Michelozzi et al. [9] had FN function improvement without decompression, while the other reported cases underwent surgery with nearly the same outcome. The small number of reported cases cannot give a clue about the role of early FN decompression to improve the FN function.

Knowledge of the blood supply of the FN and its anatomical variation is of high importance to decrease the risk of post-embolization paralysis [3,9]. The temporal part of the FN (tympanic and mastoid segments) receives blood supply from the stylomastoid artery and the petrosal branch of the middle meningeal artery (MMA). The stylomastoid artery is a branch of the occipital artery in 60%, while in 40% of patients it is a branch of the posterior auricular artery. The MMA does not supply the FN in 10%, which makes this sector of patients vulnerable to FN paralysis after embolization—especially if the stylomastoid artery arises from the occipital artery, which is usually embolized in JPs [8]. However, in some cases, FN paralysis could happen even without the presence of vascular anomalies when Onyx migrates to all arteries supplying the FN [3].

When embolization of vessels involved in the blood supply of a cranial nerve is required, using a dual-lumen balloon catheter to prevent the flow of Onyx fragments proximally with slow injection rate or using other temporary occluding agents like polyvinyl alcohol with high recanalization rate were found to be useful in decreasing the risk of nontarget vessel occlusion and neural ischemia with permanent cranial nerves deficits [9,10]. Furthermore, direct percutaneous injection of Onyx inside the tumor is another alternative route to the transarterial one, with no reported cases of post-embolization neuropathy; however, the safety of this technique is not well-established, as few cases of JPs have been embolized using this procedure [5,10].

## 4. Conclusions

Embolization with Onyx can effectively reduce the high vascularity of jugular paragangliomas with multiple properties over other embolic agents; however, there is a potential risk of facial nerve paralysis which should be discussed with patients. Awareness of vascular anomalies, caution near dangerous anastomosis, direct percutaneous injection technique, and using balloon inflation technique for dangerous vessels has been proposed to decrease the risk of post-embolization facial nerve paralysis.

## Figures and Tables

**Table 1:** Reported jugular paraganglioma cases with post-Onyx facial nerve paralysis.

Author, Year	FN H-B Grade Post-Emb.	FN H-B Grade Final	Follow-Up (Months)	Management	LCN Paralysis
Gartrell et al., 2012 [3] *	2 cases				2 cases
	1. VI	1. VI	3	Surgery	1. X, XII
	2. V	2. II	19	Surgery	2. X, XI
Gaynor et al., 2014 [8]	VI	VI	6	Surgery	X paralysis
Ladner et al., 2014 [10]	V	I	6	Surgery	None
Kadakia et al., 2015 [5]	VI		No follow-up	Observation	None
Michelozzi et al., 2016 [9] **	4 cases				4 cases
	1. III	1. I	12	Observation	3 cases X
	2. V	2. II	10		
	3. II	3. I	15		1 case X, XII
	4. IV	4. II	12		
Our case	V	II	30	Surgery	None

* Reported 3 cases however one case was carotid body tumor; ** One case had FN paralysis and one case had X, XII were aggravated after embolization. FN H-B: facial nerve House–Brackmann; Emb: embolization; LCN: lower cranial nerves.
